# Progressive MRI brain volume changes in ovine models of CLN5 and CLN6 neuronal ceroid lipofuscinosis

**DOI:** 10.1093/braincomms/fcac339

**Published:** 2023-01-02

**Authors:** Samantha J Murray, Mustafa M Almuqbel, Simon A Felton, Nickolas J Palmer, Daniel J Myall, Reza Shoorangiz, Arsène Ella, Matthieu Keller, David N Palmer, Tracy R Melzer, Nadia L Mitchell

**Affiliations:** Faculty of Agriculture and Life Sciences, Lincoln University, Lincoln 7647, New Zealand; Pacific Radiology Group, Christchurch 8014, New Zealand; New Zealand Brain Research Institute, Christchurch 8011, New Zealand; Pacific Radiology Group, Christchurch 8014, New Zealand; Pacific Radiology Group, Christchurch 8014, New Zealand; New Zealand Brain Research Institute, Christchurch 8011, New Zealand; New Zealand Brain Research Institute, Christchurch 8011, New Zealand; UMR Physiologie de la Reproduction & des Comportements, INRAE/CNRS/University of Tours, F-37380 Nouzilly, France; UMR Physiologie de la Reproduction & des Comportements, INRAE/CNRS/University of Tours, F-37380 Nouzilly, France; Faculty of Agriculture and Life Sciences, Lincoln University, Lincoln 7647, New Zealand; New Zealand Brain Research Institute, Christchurch 8011, New Zealand; Department of Medicine, University of Otago, Christchurch 8011, New Zealand; School of Psychology, Speech and Hearing, University of Canterbury, Christchurch 8041, New Zealand; Faculty of Agriculture and Life Sciences, Lincoln University, Lincoln 7647, New Zealand

**Keywords:** MRI, Batten disease, sheep, neuroanatomy, neurodegeneration

## Abstract

Neuronal ceroid lipofuscinoses (Batten disease) are a group of inherited lysosomal storage disorders characterized by progressive neurodegeneration leading to motor and cognitive dysfunction, seizure activity and blindness. The disease can be caused by mutations in 1 of 13 ceroid lipofuscinosis neuronal (CLN) genes. Naturally occurring sheep models of the CLN5 and CLN6 neuronal ceroid lipofuscinoses recapitulate the clinical disease progression and post-mortem pathology of the human disease. We used longitudinal MRI to assess global and regional brain volume changes in CLN5 and CLN6 affected sheep compared to age-matched controls over 18 months. In both models, grey matter volume progressively decreased over time, while cerebrospinal fluid volume increased in affected sheep compared with controls. Total grey matter volume showed a strong positive correlation with clinical scores, while cerebrospinal fluid volume was negatively correlated with clinical scores. Cortical regions in affected animals showed significant atrophy at baseline (5 months of age) and progressively declined over the disease course. Subcortical regions were relatively spared with the exception of the caudate nucleus in CLN5 affected animals that degenerated rapidly at end-stage disease. Our results, which indicate selective vulnerability and provide a timeline of degeneration of specific brain regions in two sheep models of neuronal ceroid lipofuscinoses, will provide a clinically relevant benchmark for assessing therapeutic efficacy in subsequent trials of gene therapy for CLN5 and CLN6 disease.

## Teaser

Murray *et al*. assessed brain volume changes in naturally occurring sheep models of neuronal ceroid lipofuscinosis using MRI. Diseased sheep showed progressive decline in overall brain volume driven by loss of grey matter and more severe atrophy in cortical regions compared to subcortical structures.

## Graphical Abstract

**Graphical Abstract:**
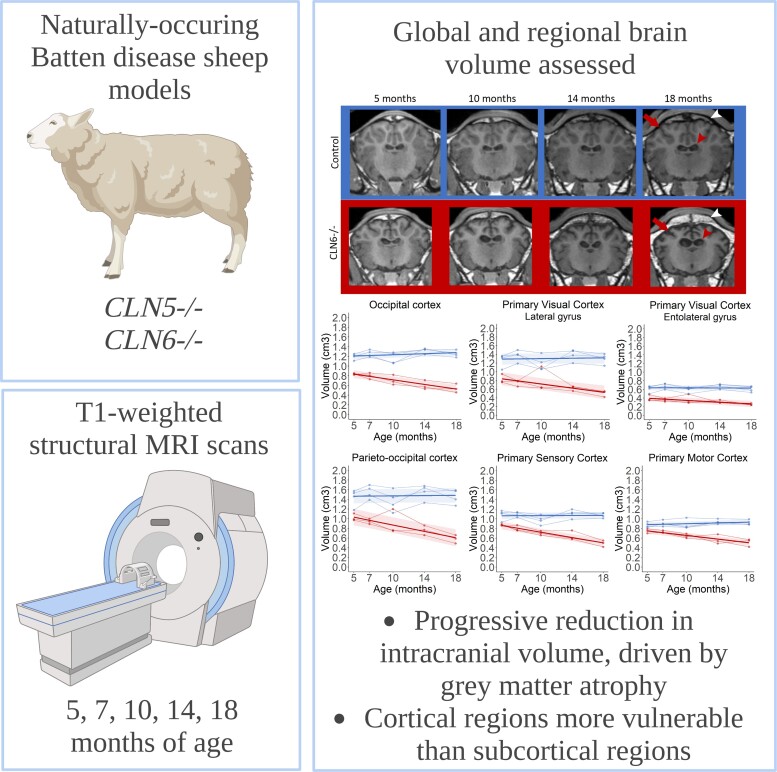

## Introduction

Neuronal ceroid lipofuscinoses (NCL; Batten disease) are a group of inherited neurodegenerative diseases primarily affecting children. There are currently 13 known variants of NCL caused by mutations in different genes now identified as *CLN1-8* and *CLN10-14* (www.ucl.ac.uk/ncl).^1^ The cellular pathological hallmark of NCL is an accumulation of proteinaceous storage material in the lysosomes of cells throughout the body, but most significantly in the brain, the major proteins stored being the c subunit of mitochondrial adenosine triphosphate (ATP) synthase or the sphingolipid activator proteins A and D.^2,3^ There is also widespread brain atrophy and neuroinflammation, resulting in motor and cognitive decline, seizures, blindness and premature death.^4^

Flocks with naturally occurring CLN5 and CLN6 variant late-infantile NCLs are established in Borderdale and South Hampshire sheep, respectively, at Lincoln University in New Zealand.^5,6^ Affected sheep display the clinicopathological features of human NCL, notably progressive blindness, a decline in mentation, cortical atrophy resulting in a reduction in brain volume, neuroinflammation and accumulation of lysosomal storage bodies.^7–9^ Clinical disease initially presents with signs of vision loss at 6 months of age, worsening motor and cognitive deficits from 12 months of age, and by end-stage disease between 18 and 24 months of age, affected sheep are blind with a severe neurological phenotype.^7,10–13^ Systematic neuropathological studies in CLN5 and CLN6 affected sheep have shown regional differences in degeneration, with the earliest and most significant neuronal loss beginning in the brain regions most prominently associated with clinical symptoms (the parieto-occipital and visual cortices) before spreading across the cerebral cortex.^9^ In contrast, the cerebellum and subcortical structures are relatively spared from disease-related pathology.

Longitudinal in-vivo assessment of intracranial volume (ICV) in CLN5 and CLN6 sheep has previously been performed using computed tomography (CT) to study the onset and progression of neurodegeneration in NCL sheep.^14^ Data obtained by 3D reconstruction of the intracranial space showed initial increases in ICV in NCL sheep followed by progressive decline in volume from 5 months of age until end-stage disease.^14^

The ICV data obtained from CT scanning were informative as an indirect measure of brain size, but this imaging modality does not allow for precise assessment of global or regional brain volumes. In contrast, MRI provides clearer delineation between grey matter (GM), white matter (WM) and cerebrospinal fluid (CSF). MRI has been used to monitor longitudinal changes in brain volume in NCL patients and is a common outcome measure used in human clinical trials to track efficacy of treatments.^15–18^ Findings from MRI studies in NCL patients include global cortical atrophy, cerebellar atrophy, increased ventricular volume and thalamic hypointensity.^15–20^ MRI is increasingly being used in large animal models of disease, including sheep,^21–23^ and interpretation of ovine MRI has been accelerated by the creation of healthy sheep brain MRI templates and both cortical and subcortical atlases for several sheep breeds.^24–27^

This study aimed to use structural MRI to track both global and regional brain volume changes over the course of ovine CLN5 and CLN6 diseases. Affected CLN5 and CLN6 sheep were scanned along with age-matched healthy controls at 5, 7, 10, 14 and 18 months of age. Volumes of GM, WM and CSF were extracted from T1-weighted scans, as well as volumes of 12 cortical and subcortical structures. These specific regions were selected *a priori* to represent the differential patterns of degeneration seen in previous neuropathological studies .^9^

## Materials and methods

### Animals

Borderdale and South Hampshire sheep were diagnosed at birth^5,6^ and maintained on the Lincoln University research farm under US National Institutes of Health guidelines for the care and use of animals in research and the NZ Animal Welfare Act (1999). All experimental protocols were approved by the Lincoln University Animal Ethics Committee. Three heterozygous control (*CLN5*^+/−^), four affected (*CLN5*^−/−^) Borderdale ewes, four heterozygous control (*CLN6*^+/−^) and three affected (*CLN6*^−/−^) South Hampshire ewes were used in the current study. Sample size was determined based on analysis methods and effect sizes seen in previous CT imaging work.^14^

### Clinical scoring

All animals were assessed monthly using the ovine Batten disease rating scale (oBDRS), which involves scoring across ten domains including vision, audition, cognition, proprioception, motor function and body condition.^7^ Healthy control sheep consistently achieve the highest score of 40, while affected sheep scores decline progressively over the course of the disease (Supplementary Fig. 1).

### MRI scanning

Sheep were scanned at 5, 7, 10, 14 and 18 months of age using a MAGNETOM Skyra 3 Tesla MRI scanner (Siemens Healthcare, Erlangen, Germany), with a 20-channel head coil. At each timepoint, sheep were scanned over a period of three weeks based on birth order. Scanning personnel were blinded to genotype.

On arrival at the scanning facility, sheep were sedated by intravenous injection of 0.5 mg/kg live weight diazepam (Troy Laboratories NZ Pty Ltd, Auckland, NZ) and 10 mg/kg live weight ketamine (Phoenix Pharm Distributors Ltd, Auckland, NZ) prior to endotracheal intubation and maintenance on inhalation anaesthesia (isoflurane in oxygen, 1.5–3% v/v to effect).

Structural imaging was performed to measure tissue volumes using a T1-weighted magnetization-prepared rapid gradient-echo sequence: echo time/repetition time = 2.02/2500 ms, inversion time = 900 ms, flip angle = 12deg, Field of view = 192 mm, slice thickness = 0.75 mm, matrix = 256 × 256, voxel size = 0.75 × 0.75 × 0.75 mm^3^, NEX = 4, scan time =25:32 min and slice acceleration GRAPPA = 2.

### MRI processing

All sheep were given unique blinding codes prior to data processing and statistical analysis. Statistical parametric mapping (SPM8; http://www.fil.ion.ucl.ac.uk/spm) in Matlab R2015a (v 8.5.0.197613, Mathworks, Massachusetts, USA) was used to perform unified segmentation and normalization of each T1-weighted image.^28^ T1 images for each sheep at each time point were roughly reoriented (manually) to a standardized ovine template, provided by Ella *et al*.^24^ T1 images were then segmented, modulated and normalized using the sheep-specific priors,^24^ with affine regularization set to an average sized template. Breed-specific tissue probability maps (TPMs) were subsequently created as follows: All modulated, normalized GM, WM and CSF images for all genotypes and ages were averaged by breed, resulting in Borderdale- and South Hampshire-specific TPMs. Segmentation and normalization were then repeated for all T1 images at all timepoints using the appropriate breed-specific TPMs during the unified segmentation. At each timepoint, total GM, WM, and CSF volumes were calculated; ICVs were calculated as the sum of the modulated normalized GM, WM and CSF segments. Additionally, volumes of 12 regions selected *a priori* based on post-mortem findings from affected sheep (primary motor cortex, primary sensory cortex, parieto-occipital cortex, orbital gyrus, orbitofrontal gyrus, cerebellum, entolateral gyrus, lateral gyrus, occipital lobe, caudate nucleus (CN), putamen and thalamus) were extracted using cortical and subcortical sheep atlases in standardized space.^24^

### Statistical analysis

Brain volumes were compared across time and groups using linear mixed effects regression models with *lme4* in R v4.0.5.^29,30^ Borderdale (*CLN5*^+/−^ and *CLN5*^−/−^) and South Hampshire (*CLN6*^+/−^ and *CLN6*^−/−^) sheep were analysed independently. A regression model was fit for each tissue type (GM, WM, CSF volumes) and each region of interest in order to determine the effects of group, time and group-by-time interactions on volume. A varying intercept was included per sheep. Sheep-level predictors were group (control or affected); measurement-level predictors included ICV and time from baseline (age), with appropriate interactions with groups. Correlations between oBDRS scores and brain tissue volumes were assessed using repeated measures correlation with *rmcorr* in R v4.0.5.^31^ Data were assessed for normality prior to analysis using the Shapiro–Wilk test with *pastecs* in R.^32^ One 7-month scan in one animal (1104/18, CLN5*^−/−^*) was excluded from analysis as the scan images showed severe movement artefact making volume extraction unreliable and inaccurate.

## Results

### Generation of breed specific sheep tissue probability maps

**Figure 1:**
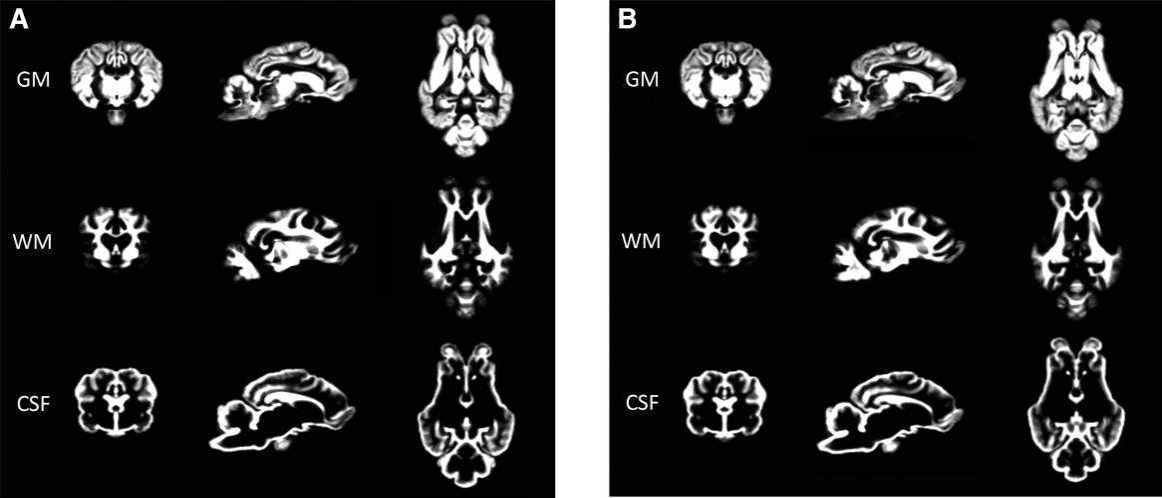
**Breed-specific TPMs.** (**A**) TPMs for GM, WM and CSF in Borderdale sheep. (**B**) TPMs for GM, WM and CSF in South Hampshire sheep.

TPMs were generated for GM, WM and CSF for Borderdale (Fig. 1A) and South Hampshire (Fig. 1B) sheep.

### Global and regional brain volume changes in healthy and CLN5 affected Borderdale sheep

**Figure 2:**
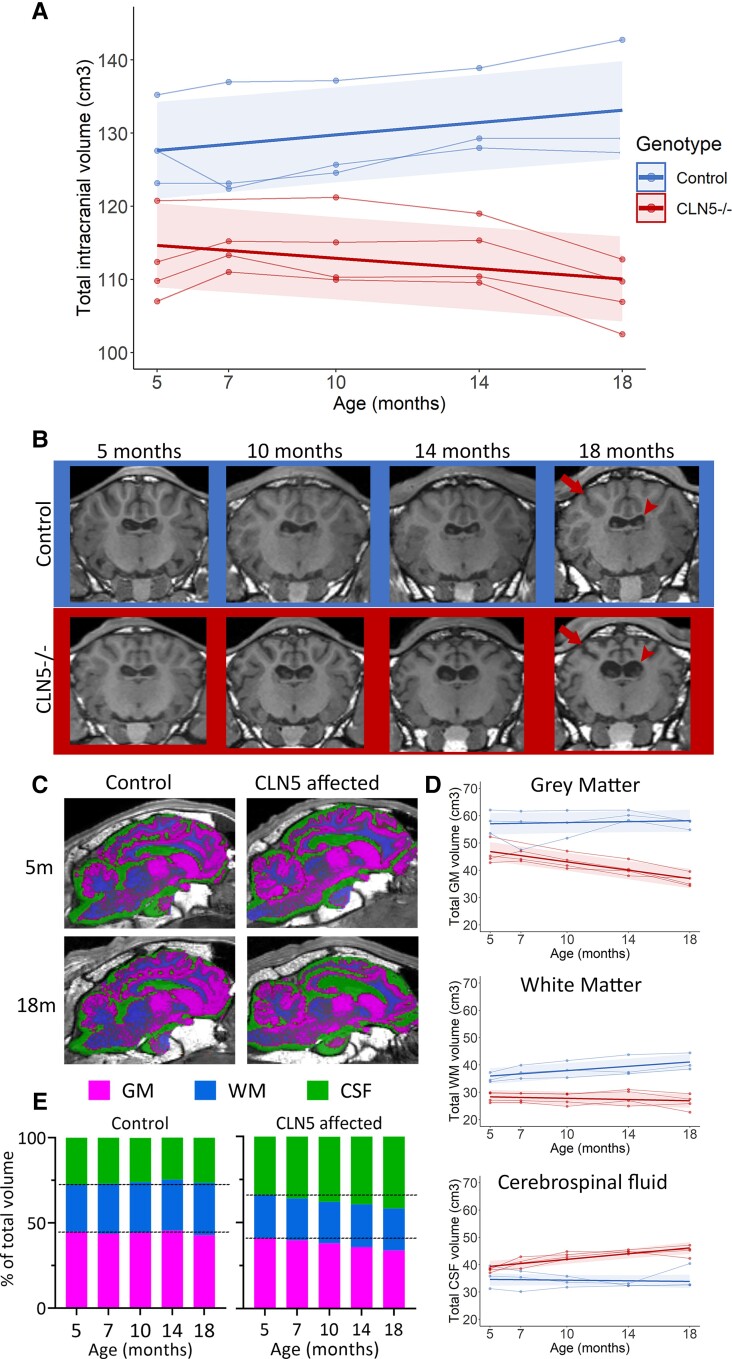
**Longitudinal ICV changes in healthy control and CLN5 affected sheep.** (**A**) ICV (cm^3^) between 5 and 18 months of age in healthy control (blue) and *CLN5*^−/−^ (red) sheep. Individual animals are represented by data points connected by thin lines, while thick lines indicate the linear regression line for each experimental group. Shaded areas indicate 95% confidence intervals of the regression model. (**B**) Coronal T1-weighted image slices from control (blue panel) and *CLN5*^−/−^ (red panel) sheep between 5 and 18 months of age. Noticeable enlarging of the lateral ventricles in *CLN5*^−/−^ sheep compared to control sheep is annotated by arrowheads, while cortical shrinkage is highlighted by arrows. (**C**) Segmented GM (magenta), WM (blue) and CSF (green) in control and *CLN5*^−/−^ sheep superimposed over T1-weighted images in coronal, sagittal and axial planes. (**D**) Analysis of volume (cm^3^) of GM, WM and CSF over time in control (blue line) and *CLN5*^−/−^ (red line) sheep. (**E**) Proportions of GM, WM and CSF at each age in control (left panel) and *CLN5*^−/−^ (right panel) sheep. Dashed lines indicate values at baseline.

Baselines scans showed a significant difference in total ICV between control and *CLN5^−/−^* sheep at 5 months of age, with the average ICV of *CLN5*^−/−^ sheep already 13% lower than controls. ICVs in the control Borderdale sheep increased by an average of 0.4cm^3^/month [95% CI (0.2, 0.7)] between 5 and 18 months of age, while the average ICV of *CLN5*^−/−^ affected sheep significantly decreased by −0.4 cm^3^/month (−0.6, −0.1) over the same period (Fig. 2A, Table 1). Representative T1-weighted images in the coronal plane show progressive widening of the lateral ventricles (Fig. 2B, arrowheads) and atrophy of the cerebral cortex (Fig. 2B, arrows) in *CLN5*^−/−^ sheep.

**Table 1:** Linear mixed effects regression model comparing rate of change in volume between control and CLN5 affected sheep

ICV	CLN5^+/−^	CLN5^−/−^	Difference
Volume at 5 m (cm^3^)	129 (114–144)	112 (103–122)	−16 (−28 to −4)
Volume at 18 m (cm^3^)	133 (112–154)	108 (101–115)	−25 (−37 to −13)
Slope (cm^3^/month)	0.4 (0.2–0.7)	−0.4 (−0.6 to −0.1)	−0.8 (−1.1 to −0.5)
**GM**	**CLN5^+/−^**	**CLN5^−/−^**	**Difference**
Volume at 5 m (cm^3^)	58 (47–58)	46 (39–53)	−12 (−20 to −3)
Volume at 18 m (cm^3^)	57 (52–61)	36 (33–40)	−20 (−25, −16)
Slope (cm^3^/month)	0.09 (−0.1 to 0.3)	−0.8 (−1 to −0.5)	−0.8 (−1.1 to −0.5)
**WM**	**CLN5^+/−^**	**CLN5^−/−^**	**Difference**
Volume at 5 m (cm^3^)	35 (31–40)	28 (25–31)	−7 (−11 to −3)
Volume at 18 m (cm^3^)	41 (33–49)	26 (22–31)	−15 (−20 to −9)
Slope (cm^3^/month)	0.4 (0.3–0.5)	−0.1 (−0.2 to 0.1)	−0.5 (−0.7 to −0.3)
**CSF**	**CLN5^+/−^**	**CLN5^−/−^**	**Difference**
Volume at 5 m (cm^3^)	36 (25–46)	38 (37–29)	2.5 (−3 to 8)
Volume at 18 m (cm^3^)	35 (24–46)	45 (42–48)	10 (4–16)
Slope (cm^3^/month)	−0.05 (−0.3 to 0.2)	0.5 (0.2–0.7)	0.6 (0.3–0.9)

Numbers in brackets indicate the 95% confidence intervals.

Both volume difference and slope difference (column 4) are considered significant if the 95% confidence interval does not include zero.

The average ICV was 19% lower in *CLN5*^−/−^ sheep compared to control sheep at 18 months of age (Fig. 2A). To determine what was driving this change, raw volumes and proportions of total ICV of segmented GM, WM and CSF were assessed. The average GM volume was 36% lower in affected sheep while the average CSF volume was 28% higher than healthy control sheep. There was very little change in GM volume from baseline to 18 months of age in control sheep while a significant decrease of 0.8 cm^3^/month on average occurred in *CLN5*^−/−^ sheep (Fig. 2C–E, Table 1). Conversely, WM remained stable [−0.1 cm^3^/month (−0.2, 0.1)] in *CLN5*^−/−^ sheep whereas it increased [0.4 cm^3^/month (0.3, 0.5)] between 5 and 18 months in control sheep. CSF volume significantly increased by an average of 0.5 cm^3^/month in *CLN5*^−/−^ sheep between baseline and 18 months of age (Fig. 2C–E, Table 1) but remained stable in control sheep. Analysis of the ratios of GM, WM, and CSF to total brain tissue confirmed these findings. There was little change in tissue proportions in control sheep, while in affected sheep the proportion of CSF progressively increased as the proportion of GM decreased (Fig. 2E). Analysis of accumulative change in volume provided a more precise time course of degeneration and showed GM declining in affected sheep from 10 months of age, while WM degeneration occurs later from 14 months of age (Supplementary Fig. 4).

**Figure 3:**
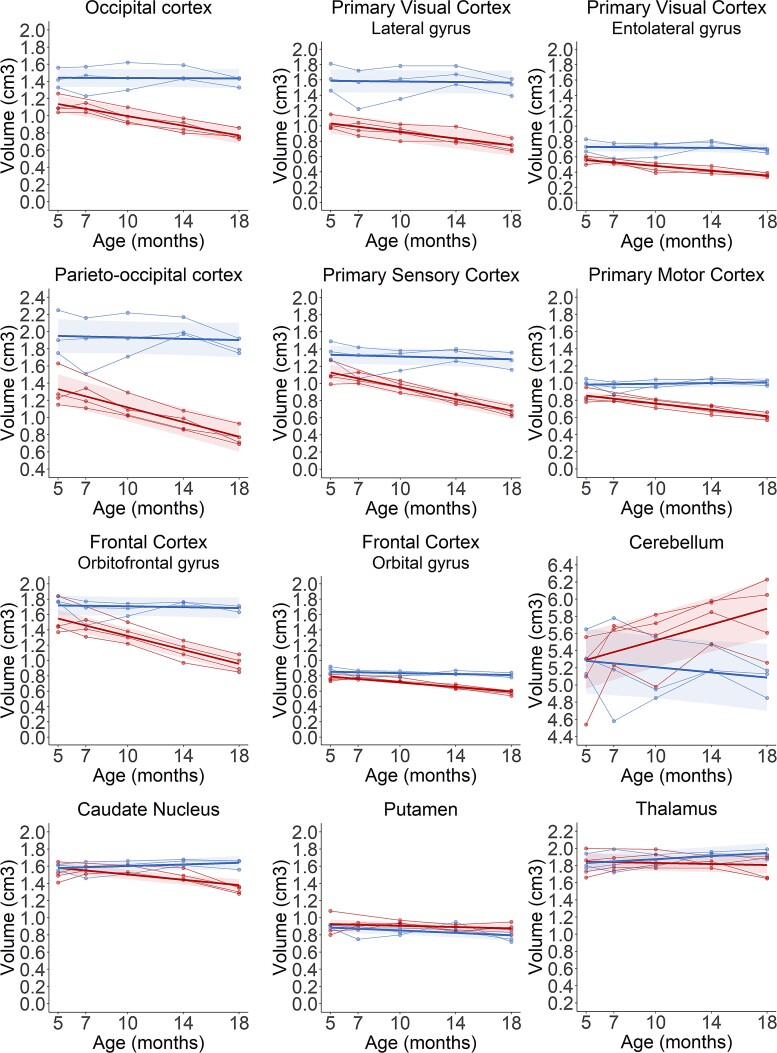
**Longitudinal regional volume changes in the brains of healthy control (blue) and CLN5 affected (red) sheep.** Individual animals are represented by data points connected by thin lines, while thick lines indicate the linear regression line for each experimental group. Shaded areas indicate 95% confidence intervals of the regression model.

Volumes of 12 cortical gyri and subcortical structures were analysed in *CLN5*^−/−^ and control sheep between baseline and 18 months of age. All of the cortical gyri analysed showed significant declines in volume in *CLN5*^−/−^ sheep over the disease course, while volumes remained relatively stable in control sheep (Fig. 3, Supplementary Table 1). The parieto-occipital and primary visual cortices were the most severely affected regions with 33 and 37% lower volumes, respectively, in *CLN5*^−/−^ sheep compared with controls at baseline. By 18 months of age these two regions were, respectively, 57 and 51% lower than controls. The primary sensory cortex also showed considerable atrophy at baseline and, similar to the parieto-occipital and primary visual cortex, began a steady decline from 7 months of age (Supplementary Fig. 5). Despite less initial atrophy in the affected orbitofrontal gyrus, this was the only region that demonstrated rapid decline beginning from baseline (Supplementary Fig. 5). Volumes in the primary sensory and orbitofrontal cortices of *CLN5*^−/−^ sheep were 47 and 43% lower than controls, respectively, at the end of the study. Cerebellar volumes were extremely variable, both within and between groups at all ages but showed no significant differences between 5 and 18 months of age. There was little difference in volumes of subcortical structures between *CLN5*^−/−^ and control sheep, with the exception of the CN which began to decline in *CLN5*^−/−^ sheep from 14 months of age (Fig. 3; Supplementary Fig. 5 and Table 1).

Linear correlations between clinical (oBDRS) scores and brain tissue volumes were assessed to determine if there was a relationship between disease stage and brain atrophy (Supplementary Fig. 2). Total GM volume showed a strong positive correlation with oBDRS scores, while CSF volume showed a strong negative correlation, indicating that as GM volume declines and CSF volume increases, clinical scores worsen. There was a moderate positive correlation between total ICV and clinical scores and only a weak correlation between WM volume and oBDRS scores (Supplementary Fig. 2).

### Global and regional brain volume changes in healthy and CLN6 affected South Hampshire sheep

**Figure 4:**
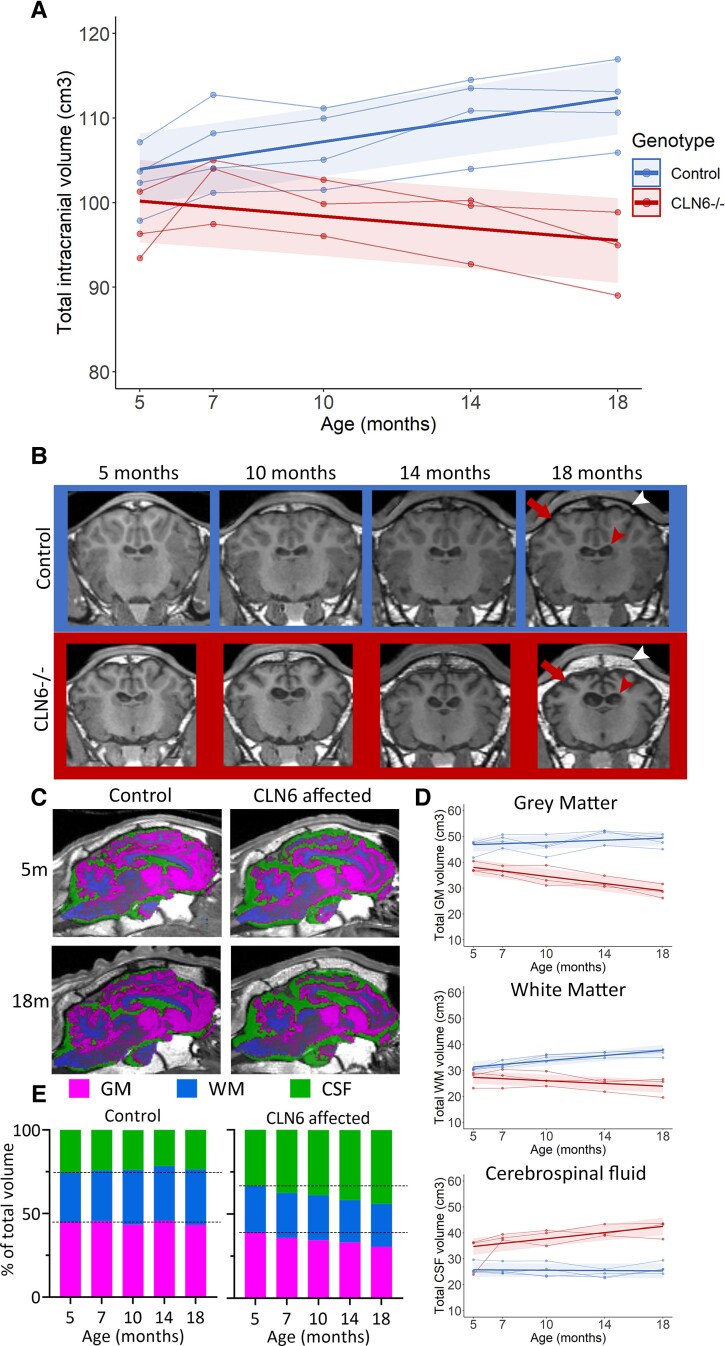
**Longitudinal ICV changes in healthy control and CLN6 affected sheep.** (**A**) ICV (cm^3^) between 5 and 18 months of in healthy control (blue) and *CLN6*^−/−^ (red) sheep. Individual animals are represented by data points connected by thin lines, while thick lines indicate the linear regression line for each experimental group. Shaded areas indicate 95% confidence intervals of the regression model. (**B**) Coronal T1-weighted image slices from control (blue panel) and *CLN6*^−/−^ (red panel) sheep between 5 and 18 months of age. Noticeable enlarging of the lateral ventricles in *CLN6*^−/−^ sheep compared to control sheep is annotated by red arrowheads, while cortical shrinkage is highlighted by arrows. White arrowheads highlight skull thickening in affected sheep compared to control sheep. (**C**) Segmented GM (magenta), WM (blue) and CSF (green) in control and *CLN6*^−/−^ sheep superimposed over T1-weighted images in coronal, sagittal and axial planes. (**D**) Analysis of volume (cm^3^) of GM, WM and CSF over time in control (blue line) and *CLN6*^−/−^ (red line) sheep. (**E**) Proportions of GM, WM and CSF at each age in control (left panel) and *CLN6*^−/−^ (right panel) sheep. Dashed lines indicate values at baseline.

At baseline, ICVs were comparable between *CLN6*^−/−^ and control sheep, and individuals in both groups demonstrated growth between 5 and 7 months of age (Fig. 4A). While the overall ICV of *CLN6*^−/−^ sheep progressively decreased by 0.4 cm^3^/month (−0.6 to −0.1) between baseline and 18 months of age healthy control South Hampshire sheep ICV continued to increase [0.7 cm^3^/month (0.4, 0.9)], over the same period (Fig. 4A, Table 2). Representative T1-weighted MR images showed an obvious widening of the *CLN6*^−/−^ lateral ventricles (Fig. 4B, red arrowheads) and severe shrinkage of the cerebral cortex (Fig. 4B, arrows), as well as significant thickening of the skull over time compared to little change in the gross anatomy of the control brain over time (Fig. 4B, white arrowheads).

**Table 2:** Linear mixed effects regression model comparing rate of change in volume between control and CLN6 affected sheep

ICV	CLN6^+/−^	CLN6^−/−^	Difference
Volume at 5 m (cm^3^)	103 (97–109)	97 (87–107)	−6 (−13 to 2)
Volume at 18 m (cm^3^)	112 (104–119)	94 (82–107)	−17 (−27 to −8)
Slope (cm^3^/month)	0.7 (0.4–0.9)	−0.4 (−0.6 to −0.1)	−1 (−1 to −0.7)
**GM**	**CLN6^+/−^**	**CLN6^−/−^**	**Difference**
Volume at 5 m (cm^3^)	46 (42–51)	38 (33–43)	−8 (−13 to −3)
Volume at 18 m (cm^3^)	48 (44–52)	29 (22–36)	−19 (−25 to −14)
Slope (cm^3^/month)	0.2 (0.04–0.3)	−0.7 (−0.9 to −0.5)	−0.9 (−1 to −0.7)
**WM**	**CLN6^+/−^**	**CLN6^−/−^**	**Difference**
Volume at 5 m (cm^3^)	30 (30–31)	27 (19–35)	−4 (−8 to 0.3)
Volume at 18 m (cm^3^)	37 (35–39)	24 (14–34)	−13 (−18 to −8)
Slope (cm^3^/month)	0.5 (0.4–0.6)	−0.3 (−0.4 to −0.1)	−0.8 (−0.9 to −0.6)
**CSF**	**CLN6^+/−^**	**CLN6^−/−^**	**Difference**
Volume at 5 m (cm^3^)	26 (23–30)	32 (15–50)	6 (−4 to 15)
Volume at 18 m (cm^3^)	26 (23–30)	41 (33, 50)	15 (10–20)
Slope (cm^3^/month)	−0.03 (−0.2 to 0.2)	0.6 (0.4–0.8)	0.6 (0.3–1)

Numbers in square brackets indicate the 95% confidence intervals.

Both volume difference and slope difference (column 4) are considered significant if the 95% confidence interval does not include zero.

The average total ICV in *CLN6*^−/−^ sheep was 16% lower than controls at 18 months of age, while the GM volume was 40% lower and CSF volume was 57% higher. From baseline, the GM volume in *CLN6*^−/−^ sheep declined significantly [−0.7 cm^3^/month, (−0.9 to −0.5)] while it increased in control sheep (0.2 cm^3^/month (0.04–0.3); Fig. 4C–E, Table 2). WM volumes followed a similar trajectory, significantly decreasing [−0.3cm^3^/month (−0.4 to −0.1)] in *CLN6*^−/−^ sheep between 5 and 18 months while increasing [0.5 cm^3^/month (0.4–0.6)] in control sheep over the same period. CSF volumes significantly increased by an average of 0.6 cm^3^/month in *CLN6*^−/−^ sheep over the study but remained stable in control sheep (Fig. 4C–E, Table 2). As in the CLN5 model, the proportion of CSF progressively increased in *CLN6*^−/−^ sheep as the proportion of GM decreased, while ratios remained constant in control sheep at all timepoints analysed (Fig. 4E). Analysis of accumulative change showed a steep decline in GM from baseline with minor decreases in WM evident from 10 months of age (Supplementary Fig. 4).

**Figure 5:**
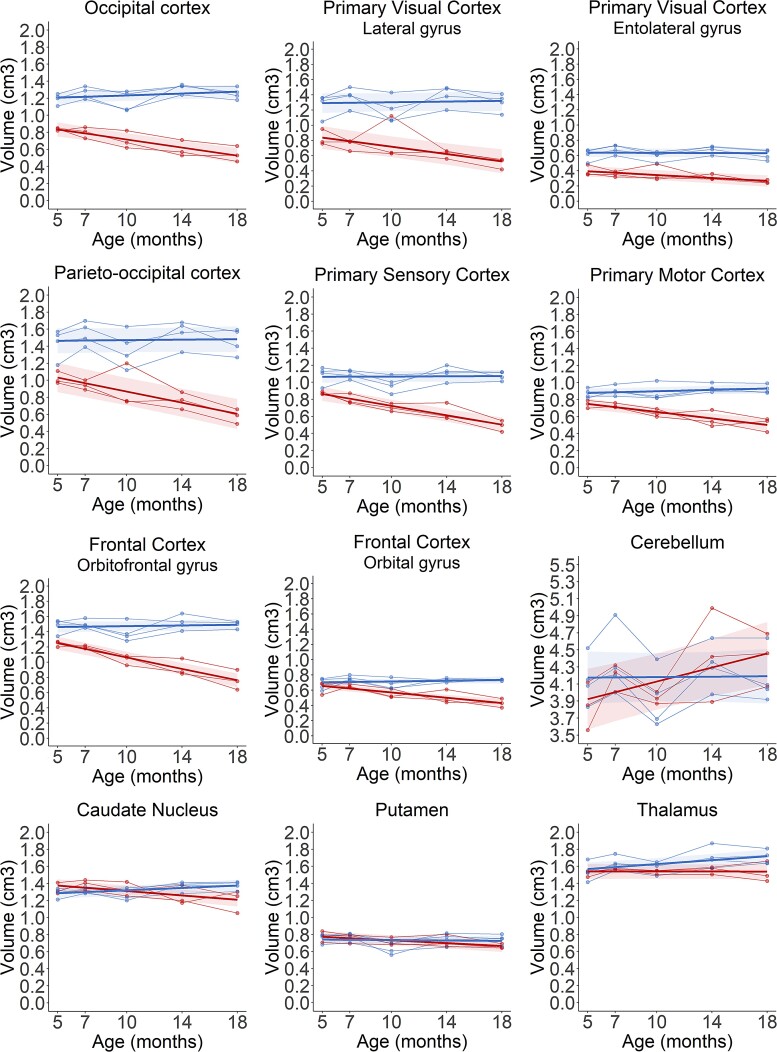
**Longitudinal regional volume changes in the brains of healthy control (blue) and CLN6 affected (red) sheep.** Individual animals are represented by data points connected by thin lines, while thick lines indicate the linear regression line for each experimental group. Shaded areas indicate 95% confidence intervals of the regression model.

The majority of the segmented cortical gyri analysed were significantly smaller in volume in *CLN6*^−/−^ sheep at baseline compared to control sheep and they all progressively declined in volume over the study (Fig. 5; Supplementary Fig. 6 and Table 2). The most marked differences at baseline were seen in the three visual areas (entolateral and lateral gyri, and the occipital cortex) which all had volumes 30–35% lower in *CLN6*^−/−^ sheep compared to controls. By end-stage disease, these regional volumes were 57–61% lower in *CLN6*^−/−^ sheep, while the parieto-occipital cortex also showed a 60% reduction compared to controls. Conversely, there were no significant age-related differences in volumes of the cerebellum and subcortical structures between *CLN6*^−/−^ and control sheep at any age.

Linear correlations between *CLN6*^−/−^ clinical (oBDRS) scores and brain tissue volumes showed positive correlations with GM, WM and total ICV (Supplementary Fig. 3). In contrast, CSF volume was negatively correlated with oBDRS score, confirming that clinical symptoms worsen as brain tissue atrophies and CSF volume increases.

## Discussion

This study utilized MRI to assess global and regional neurodegeneration in naturally occurring sheep models of CLN5 and CLN6 NCL. The detailed dataset created using this clinically relevant and non-invasive imaging technique has established a benchmark for assessment of therapeutic efficacy in trials of gene therapy in both NCL sheep models that is translatable to human medicine.

Baseline scans were performed at 5 months of age, a clinically pre-symptomatic disease stage. MRI revealed already significantly lower average ICVs in CLN5 affected sheep at this age compared to healthy controls. However, there was considerable variation between affected individuals (range 107–121 cm^3^) at this early age, indicating that a single pre-symptomatic MRI scan could not be reliably used as an early indicator of disease status. Subsequent scans were performed at early-symptomatic (7 months) and advanced-symptomatic disease stages (10 and 14 months), with the final MRI scan at end-stage disease (18 months). Throughout the period of increasing disease symptomology, ICVs in the CLN5 affected sheep failed to increase and began to decline in volume from 14 months of age. A similar pattern of growth was observed in the CLN6 affected sheep; however, brain volumes began to decline even earlier (observable at 7 months). Of note, these MRI-derived ICV changes mirrored those previously determined for CLN5 and CLN6 sheep using a CT imaging modality.^14^

MRI has a much greater range of soft tissue contrast and depicts neuroanatomy better than CT. Here, the ratio of GM, WM and CSF to total brain tissue detected by MRI provides an additional method to monitor brain changes. CLN5 and CLN6 healthy controls exhibited stable GM and CSF ratios throughout the study, with minor WM ratio increases. However, GM ratios decreased and CSF ratios increased in both CLN5 and CLN6 affected sheep over time. In contrast, the proportion of WM remained relatively constant over the course of the study. This shows that the main drivers of the ICV changes are the loss of GM and concurrent increase in CSF. In addition, GM appears to be more vulnerable to degeneration earlier and at a faster rate than WM in ovine NCL. This may be due to the initial loss of neuronal cell bodies and subsequent degeneration of axonal fibres. This is in keeping with studies of post-mortem brain tissue from NCL sheep where GM atrophy and lysosomal storage appeared earlier and was more severe than WM pathology.^9^

All cortical regions assessed showed declining volume from baseline to 18 months of age in both CLN5 and CLN6 affected animals, while the same regions in control brains maintained stable volumes. The parieto-occipital cortex showed the most severe atrophy at baseline and greatest rate of decline over the disease course. The primary visual cortex also displayed severe atrophy at baseline; however, it did not then decline as dramatically. The primary sensory and orbitofrontal cortices in affected animals were comparable to controls at baseline but then showed severe decline similar to that of the parieto-occipital cortex. The motor cortex displayed the least degeneration in affected animals. This corroborates previously published neuropathological data from both NCL sheep breeds, which showed an identical pattern of differential cortical thinning and neuroinflammation across the affected brain.^9^ The current MRI study also indicated that cerebellar and subcortical volumes were not significantly different between control and affected animals of both breeds throughout disease, with the exception of the CN which began to decline at end stage disease. This is in keeping with studies of post-mortem brain tissue from NCL sheep shows no overt atrophy or morphological deficits in the cerebellum or subcortex at end-stage disease.^9^

MRI data from patients with late-infantile NCLs are generally limited to case studies or small cohorts of individuals assessed at single time points. Despite this, consistent findings have been observed across human studies including cortical and cerebellar atrophy, basal ganglia and brain stem hyperintensity, and thalamic hypointensity.^17,19,33–36^ Cerebellar atrophy is reported to be more severe than cortical atrophy in CLN5 patients, while in CLN6 patients although the cerebellum does shrink, it is not as severe as the cortical volume loss.^19^ As mentioned above, we do not observe cerebellar degeneration in CLN5 or CLN6 affected sheep either by *in vivo* neuroimaging or post-mortem tissue analysis. The reason for the lack of cerebellar pathology in CLN5 and CLN6 sheep is unknown, however may manifest if affected sheep were studied beyond the humane endpoint of 18–24 months of age. The thalamus is another region of interest in NCL patients and animal models, as it typically displays hypointensity in patient MRI, and minor late stage atrophy has been shown in different breeds of CLN6 sheep.^17,19,23,35,37^ Although the pathomechanisms leading to thalamic hypointensity in MRI scans are unknown, Autti *et al.*^38^ postulate that decreased signal intensity from the thalamus is a biomarker of lysosomal storage disease as it is commonly seen not only in patients with NCL, but also in Tay-Sachs, Sandhoff’s disease and Krabbe’s disease. A previous study evaluated gross and regional brain volumes over time in CLN6 affected South Hampshire sheep using MRI however the baseline scan was performed at approximately 17 months of age, when animals were nearing end-stage disease.^23^ At 17 months of age, CLN6 affected sheep were reported to have 40% lower cortical volume compared to age-matched healthy controls, with sparing of the cerebellum. Subcortical structures did not show the same level of atrophy but did decline in volume between 17 and 22 months of age, with the most significant atrophy seen in the CN.^23^ In the current study we did not see this same level of atrophy in subcortical structures of CLN6 affected sheep. Although volumes of the CN, putamen and thalamus were all 10–13% lower in affected sheep compared to controls at 18 months of age, they were not significantly different. This indicates that atrophy of the subcortex is a feature of end stage disease that occurs subsequent to severe cortical degeneration. Of note, life expectancy in these NCL sheep models is typically limited to between 18 and 24 months of age. However, this is a humane terminal endpoint specified by researchers when a sheep scores ≤ 1 for the non-sensory domains of the oBDRS indicating their quality of life is dramatically reduced, or they can no longer live independently. In the current study the final MRI assessment was done at 18-months of age, a pre-determined end-point which considered animal welfare implications. Sheep had to be transported 30 minutes away for scanning procedures and there was concern that affected animals would find this increasingly stressful at later time points. The CLN5 affected sheep from this study reached a humane endpoint requiring euthanasia at 20–21 months of age, while CLN6 affected sheep were humanely euthanized at 19 months of age. This is distinct from end-stage disease in affected children who may experience long periods of palliative care many years after they can no longer function independently. This may explain some of the discrepancy in brain structural changes between sheep models and patients, particularly with regard to the cerebellum and subcortex.

The emergence of a clinical phenotype is similar between CLN5 and CLN6 affected sheep and begins with deficits such as low head carriage and baulking, crouching or stumbling when moving through gateways around 6 months of age.^7,10,12^ By 9 months of age affected sheep show reduced menace response and herding behaviour and reduced retinal function as assessed by electroretinography.^13^ Motor, sensory and cognitive deficits emerge around 12 months of age, presenting as ataxia, gait abnormalities and reduced mentation. Stereotypical behaviours such as compulsive circling become evident around 15 months of age. By 18 months of age affected sheep have no remaining retinal function and many demonstrate inducible seizure activity.^7,10,12^ The progression of neurodegeneration demonstrated here by MRI marries with the established timeline of symptomology in NCL sheep. In CLN5 affected sheep we demonstrated early atrophy of frontal and visual cortices, followed by motor and sensory cortices between 7 and 14 months of age, with all cortical regions analysed showing atrophy by 18 months of age. The cortices of CLN6 affected sheep all showed atrophy at baseline which progressed at similar rates to those of CLN5 affected sheep. Relatively little is known about the role of the frontal cortex in sheep; however, it is postulated to be involved in emotional regulation, reward encoding, learning, attention, and facial recognition.^39–42^ Given these proposed functions, the severe atrophy we have shown in the frontal cortex of Batten disease sheep makes sense in the context of the progressive cognitive decline we observe, in particular reduced ability to traverse a maze, lack of awareness of surroundings and reduced flocking behaviour.^7^ In contrast, the motor cortex shows later and less severe degeneration. Therefore, it is likely that early motor symptoms in affected sheep, such as stumbling and baulking, may be a result of visual cortex atrophy and vision loss, while motor symptoms that appear later, such as ataxia and akinesia, are likely due to subsequent degeneration of the motor and sensory cortices. There was little or no degeneration of basal ganglia structures involved in regulation of motor and limbic functions or of the thalamus which acts as a relay centre for information going to and from the cerebral cortex. In addition, the cerebellum, involved in motor co-ordination and balance, was also spared. The reason for the discrepancy in cerebellar pathology between patients and large animal models, and how this relates to symptomology is still unclear.

While previous studies of affected sheep brain tissue have demonstrated which regions are most significantly degenerated at end-stage disease,^9,43^ the current study provides a time course of this degeneration and shows that many affected cortical regions, including the primary motor, sensory and visual cortices, are already significantly atrophied before the onset of symptoms. Understanding the time course of atrophy in specific brain regions was expected to inform on potential target areas for treatment; however as we have demonstrated early and widespread atrophy throughout the affected sheep brain, this validates our approach of pre- or early-symptomatic intracerebroventricular gene therapy injections to give widespread distribution of therapeutic vector around the central nervous system.^7^ The development of breed specific TPMs will aid in providing accurate segmentation of Borderdale and South Hampshire brains in future studies where MRI will be used to assess therapeutic efficacy in pre-clinical gene therapy trials for NCL (templates are available here https://osf.io/we5kj/). This study also highlights the feasibility and reliability of regular MRI scanning of sheep models of neurodegenerative disease. Further investigation is required into WM integrity and regions of hyper- or hypointensity on T2-weighted scans in CLN5 and CLN6 affected sheep, and studies are underway to assess these parameters using high angular resolution diffusion imaging and T2-weighted scans. Assessment of volume changes in cortical and subcortical regions in NCL sheep has added to our understanding of the selective vulnerability and timeline of degeneration of specific brain regions. This data will also inform on regions of interest for monitoring in current clinical trials of gene therapy for CLN5 (Clinicaltrials.gov NCT05228145) and CLN6 (Clinicaltrials.gov NCT02725580) NCL in humans that are using MRI as an outcome measure for assessing therapeutic efficacy.

## Supplementary Material

fcac339_Supplementary_Data

## Data Availability

TPMs generated for Borderdale and South Hampshire sheep are available at the Centre for Open Science; https://osf.io/we5kj/.

## Abbreviations

CN =: caudate nucleus
CSF =: cerebrospinal fluid
CT =: computed tomography
GM =: grey matter
ICV =: intracranial volume
NCL =: neuronal ceroid lipofuscinoses (Batten disease)
oBDRS =: ovine Batten disease rating scale
TPM =: tissue probability map
WM =: white matter
