# Supplementary material for: Progressive MRI brain volume changes in ovine models of CLN5 and CLN6 neuronal ceroid lipofuscinosis
Source: Brain Commun. 2023 Jan 2;5(1):fcac339. doi: 10.1093/braincomms/fcac339 (PMC9830986; doi:10.1093/braincomms/fcac339)
Supplement: fcac339_Supplementary_Data [file fcac339_supplementary_data.pdf]

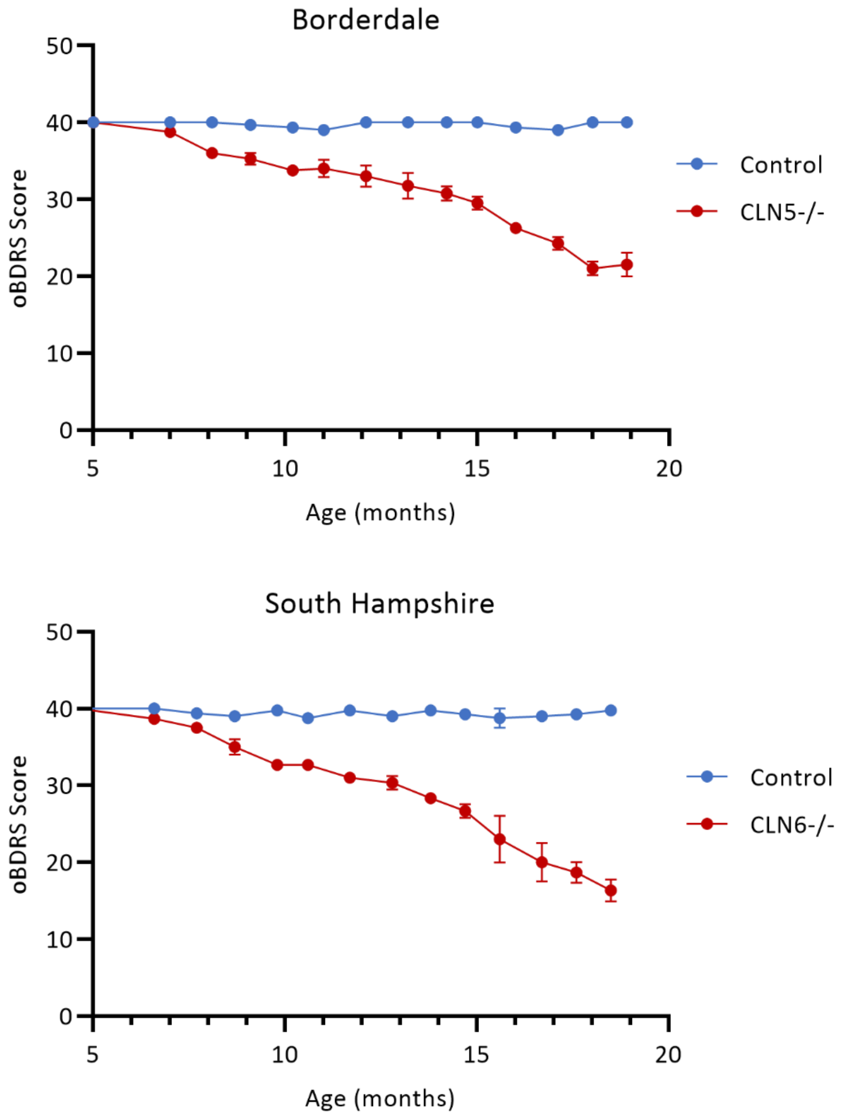


**Supplementary Figure 1.** Mean ± SEM ovine Batten disease rating scale scores in control (blue) and affected (red) animals of each breed.

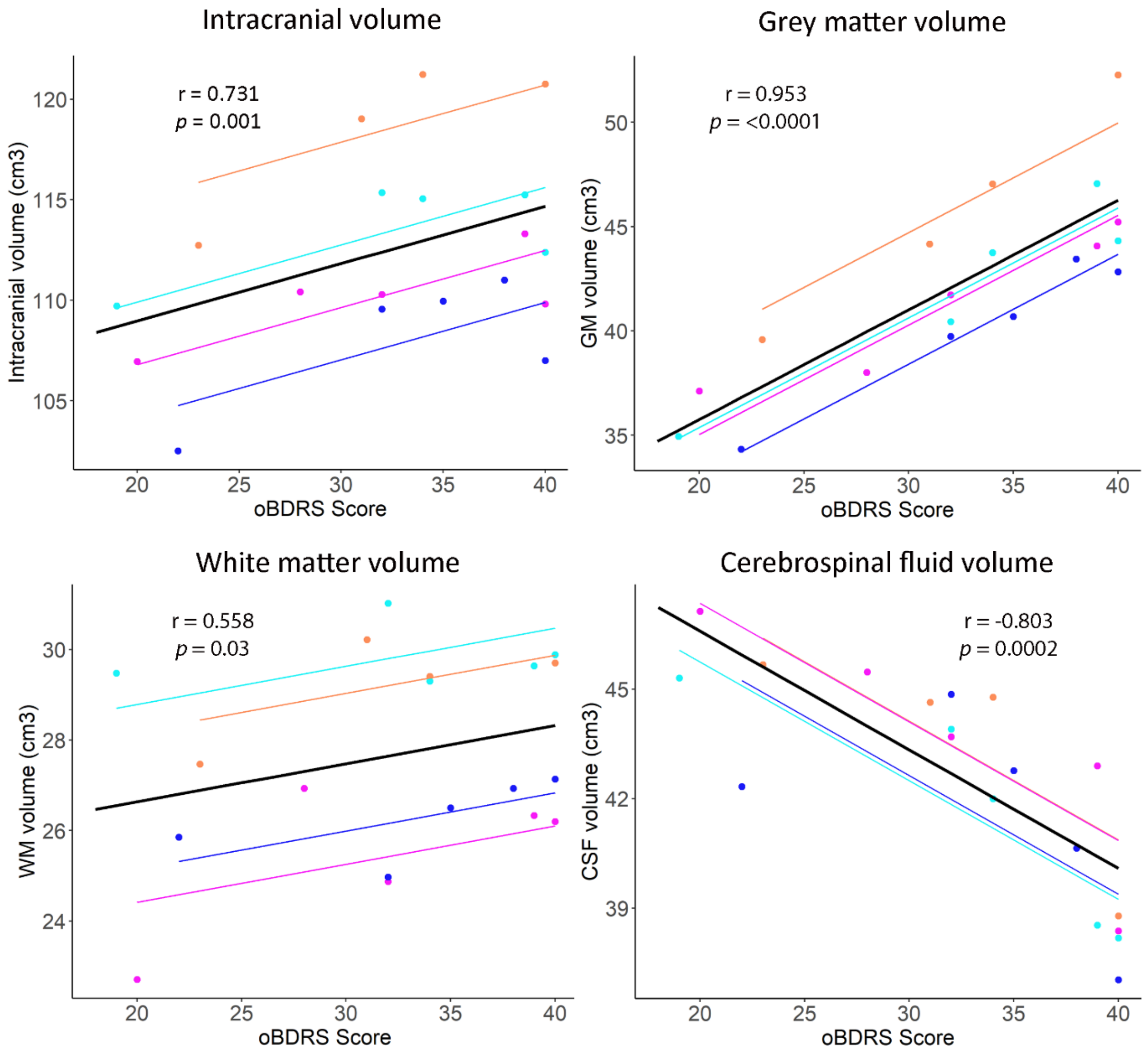


**Supplementary Figure 2.** Correlation between oBDRS score and total intracranial (ICV), grey matter (GM), white matter (WM) and cerebrospinal fluid (CSF) volumes in Borderdale sheep. Overall correlations are denoted by the black line, while repeated measures in individual animals are denoted by the coloured points and lines.

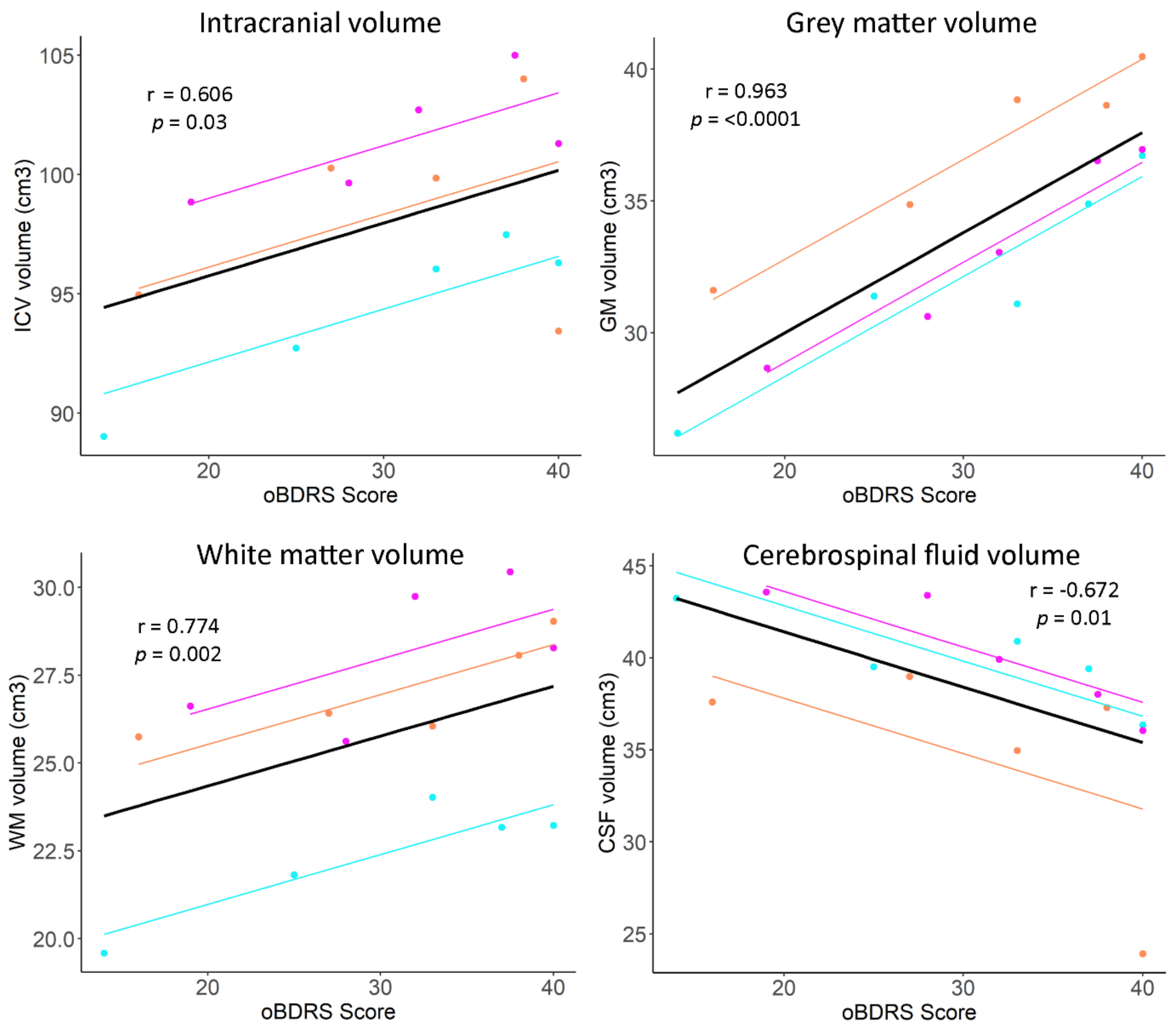


**Supplementary Figure 3.** Correlation between oBDRS score and total intracranial (ICV), grey matter (GM), white matter (WM) and cerebrospinal fluid (CSF) volumes in South Hampshire sheep. Overall correlations are denoted by the black line, while repeated measures in individual animals are denoted by the coloured points and lines.

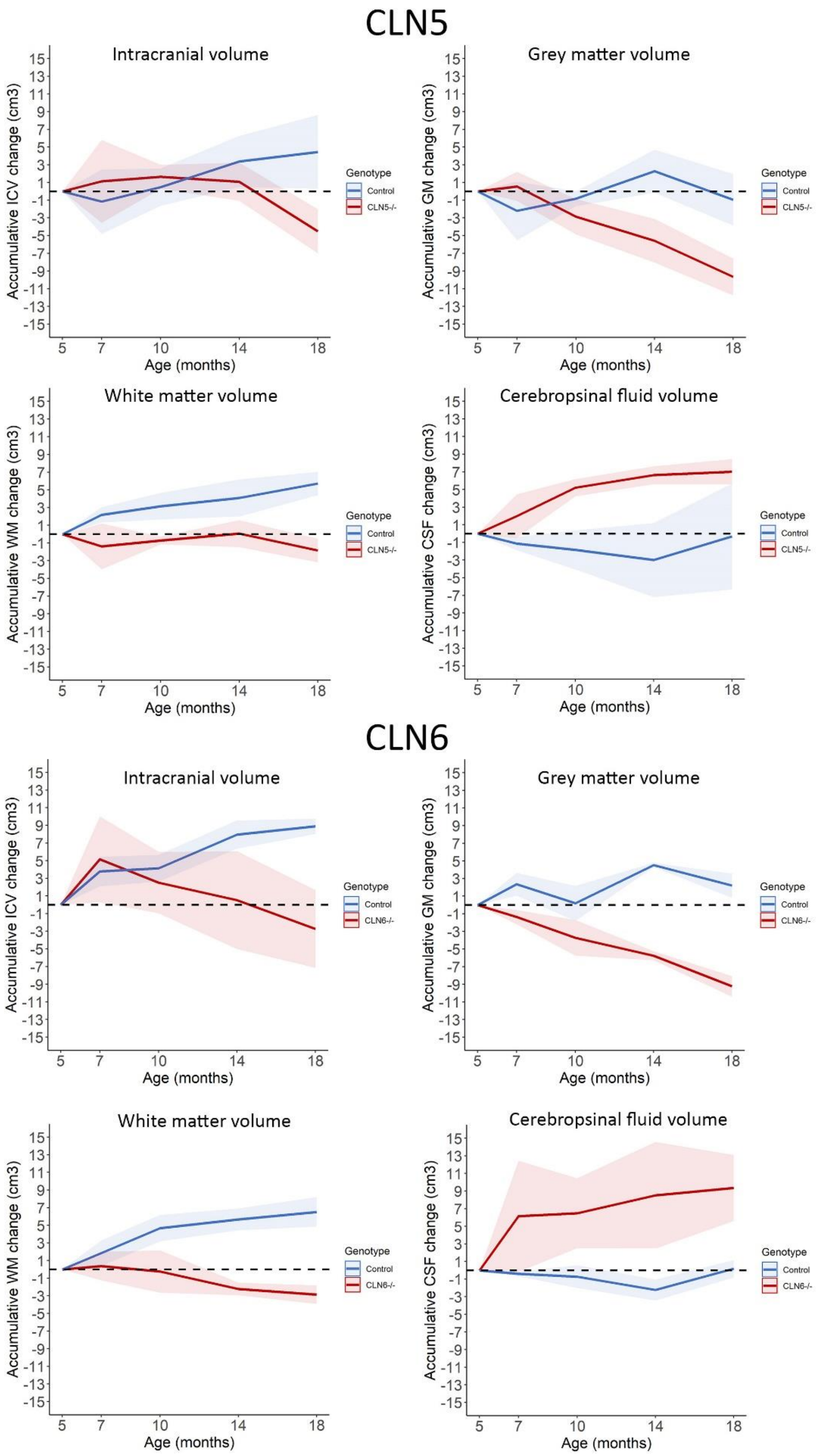


**Supplementary Figure 4.** Mean (± SD) accumulative change of intracranial (ICV), grey matter (GM), white matter (WM) and cerebrospinal fluid (CSF) in control (blue) and affected (red) sheep. Dashed black line indicates zero.

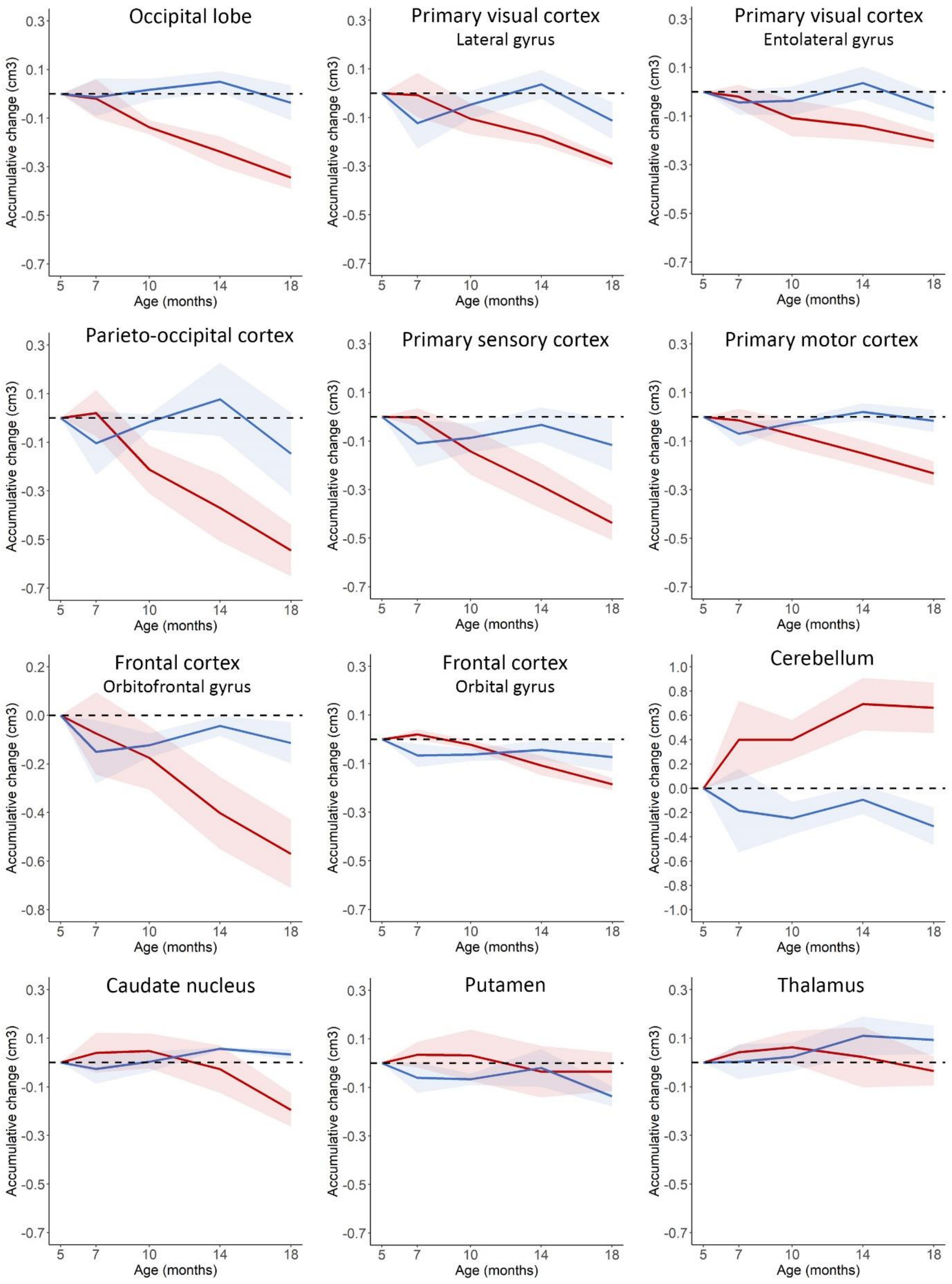


**Supplementary Figure 5.** Mean (± SD) accumulative change of cortical and subcortical brain regions in CLN5 +/- (control; blue) and CLN5-/- (affected; red) sheep. Dashed black line indicates zero.

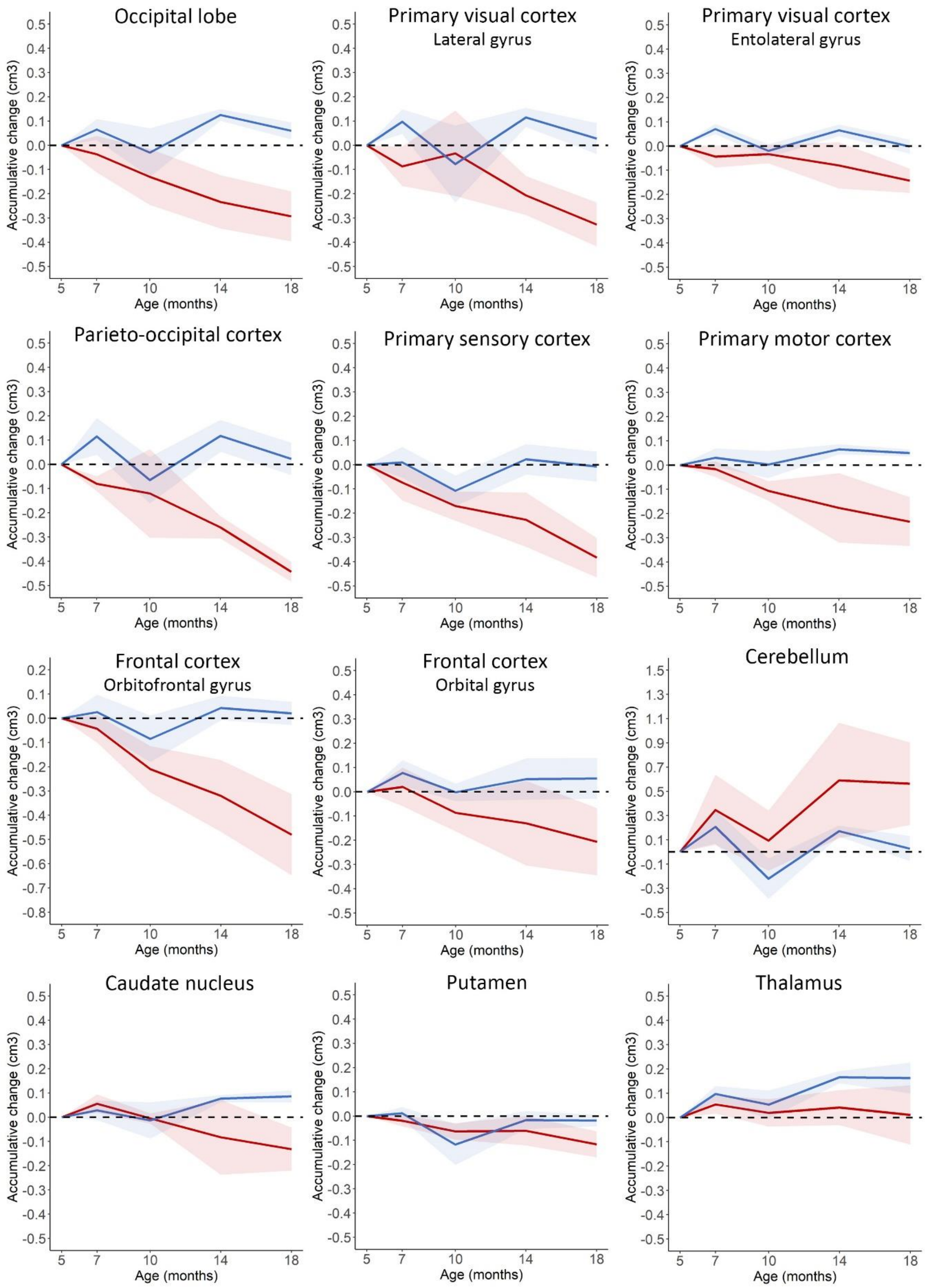


**Supplementary Figure 6.** Mean (± SD) accumulative change of cortical and subcortical brain regions in CLN6 +/- (control; blue) and CLN6-/- (affected; red) sheep. Dashed black line indicates zero.

**Supplementary Table 1. Linear mixed effects regression model comparing rate of change in volume between control and CLN5 affected sheep.**

| **Primary motor cortex** | **CLN5$^{+/-}$** | **CLN5$^{-/-}$** | **Difference** |
|---|---|---|---|
| Volume at 5m (cm$^3$) | 1<br>[0.9, 1.1] | 0.8<br>[0.7, 1] | -0.2<br>[-0.3, -0.05] |
| Volume at 18m (cm$^3$) | 1<br>[0.9, 1.1] | 0.6<br>[0.6, 0.7] | -0.4<br>[-0.5, -0.3] |
| Slope (cm$^3$/month) | 0.002<br>[-0.002, 0.006] | -0.02<br>[-0.02, -0.01] | -0.02<br>[-0.03, -0.01] |
| **Primary sensory cortex** | **CLN5$^{+/-}$** | **CLN5$^{-/-}$** | **Difference** |
| Volume at 5m (cm$^3$) | 1.4<br>[1.1, 1.6] | 1.1<br>[0.9, 1.3] | -0.3<br>[-0.5, -0.05] |
| Volume at 18m (cm$^3$) | 1.3<br>[1, 1.5] | 0.7<br>[0.6, 0.8] | -0.6<br>[-0.7, -0.4] |
| Slope (cm$^3$/month) | -0.004<br>[-0.01, 0.002] | -0.03<br>[-0.04, -0.03] | -0.03<br>[-0.04, -0.02] |
| **Parieto-occipital cortex** | **CLN5$^{+/-}$** | **CLN5$^{-/-}$** | **Difference** |
| Volume at 5m (cm$^3$) | 2<br>[1.3, 2.6] | 1.3<br>[1, 1.7] | -0.6<br>[-1.1, -0.2] |
| Volume at 18m (cm$^3$) | 1.8<br>[1.6, 2] | 0.8<br>[0.6, 0.9] | -1<br>[-1.2, -0.8] |
| Slope (cm$^3$/month) | -0.004<br>[-0.01, 0.008] | -0.04<br>[-0.05, -0.03] | -0.04<br>[-0.05, -0.02] |
| **Orbital gyrus** | **CLN5$^{+/-}$** | **CLN5$^{-/-}$** | **Difference** |
| Volume at 5m (cm$^3$) | 0.9<br>[0.8, 1] | 0.8<br>[0.7, 0.8] | -0.1<br>[-0.2, -0.04] |
| Volume at 18m (cm$^3$) | 0.8<br>[0.7, 0.9] | 0.6<br>[0.5, 0.6] | -0.2<br>[-0.3, -0.2] |
| Slope (cm$^3$/month) | -0.003<br>[-0.007, 0.0007] | -0.02<br>[-0.02, -0.01] | -0.01<br>[-0.02, -0.01] |
| **Orbitofrontal gyrus** | **CLN5$^{+/-}$** | **CLN5$^{-/-}$** | **Difference** |
| Volume at 5m (cm$^3$) | 1.8<br>[1.7, 1.9] | 1.5<br>[1.2, 1.9] | -0.3<br>[-0.6, 0.06] |
| Volume at 18m (cm$^3$) | 1.7<br>[1.6, 1.8] | 1<br>[0.8, 1.1] | -0.7<br>[-0.9, -0.6] |
| Slope (cm$^3$/month) | -0.003<br>[-0.01, 0.006] | -0.05<br>[-0.05, -0.04] | -0.04<br>[-0.05, -0.03] |
| **Cerebellum** | **CLN5$^{+/-}$** | **CLN5$^{-/-}$** | **Difference** |
| Volume at 5m (cm$^3$) | 5.4<br>[4.7, 6.0] | 5.1<br>[4.4, 5.8] | -0.2<br>[-1, 0.5] |
| Volume at 18m (cm$^3$) | 5.1<br>[4.6, 5.5] | 5.8<br>[5.1, 6.5] | 0.7<br>[0.04, 1.4] |
| Slope (cm$^3$/month) | -0.02<br>[-0.04, 0.008] | 0.05<br>[0.03, 0.07] | 0.06<br>[0.03, 0.09] |
| **Entolateral gyrus** | **CLN5$^{+/-}$** | **CLN5$^{-/-}$** | **Difference** |

| | | | |
|---|---|---|---|
| Volume at 5m ($cm^3$) | 0.7<br>[0.5, 0.9] | 0.6<br>[0.5, 0.6] | -0.2<br>[-0.3, -0.05] |
| Volume at 18m ($cm^3$) | 0.7<br>[0.6, 0.7] | 0.4<br>[0.3, 0.4] | -0.3<br>[-0.4, -0.3] |
| Slope ($cm^3$/month) | -0.002<br>[-0.007, 0.004] | -0.02<br>[-0.02, -0.01] | -0.01<br>[-0.02, -0.01] |
| **Lateral gyrus** | **$CLN5^{+/-}$** | **$CLN5^{-/-}$** | **Difference** |
| Volume at 5m ($cm^3$) | 1.6<br>[1.2, 2.1] | 1<br>[0.9, 1.2] | -0.6<br>[-0.9, -0.3] |
| Volume at 18m ($cm^3$) | 1.5<br>[1.2, 1.8] | 0.7<br>[0.6, 0.9] | -0.8<br>[-1, -0.6] |
| Slope ($cm^3$/month) | -0.002<br>[-0.009, 0.006] | -0.02<br>[-0.03, -0.02] | -0.02<br>[-0.03, -0.01] |
| **Occipital lobe** | **$CLN5^{+/-}$** | **$CLN5^{-/-}$** | **Difference** |
| Volume at 5m ($cm^3$) | 1.4<br>[1.1, 1.7] | 1.1<br>[1, 1.3] | -0.3<br>[-0.1, -0.5] |
| Volume at 18m ($cm^3$) | 1.4<br>[1.2, 1.6] | 0.8<br>[0.7, 0.9] | -0.6<br>[-0.7, -0.5] |
| Slope ($cm^3$/month) | -0.0006<br>[-0.006, 0.005] | -0.03<br>[-0.03, -0.02] | -0.03<br>[-0.03, -0.02] |
| **Caudate nucleus** | **$CLN5^{+/-}$** | **$CLN5^{-/-}$** | **Difference** |
| Volume at 5m ($cm^3$) | 1.6<br>[1.5, 1.7] | 1.5<br>[1.4, 1.7] | -0.08<br>[-0.2, 0.08] |
| Volume at 18m ($cm^3$) | 1.6<br>[1.5, 1.8] | 1.3<br>[1.3, 1.4] | -0.3<br>[-0.4, -0.2] |
| Slope ($cm^3$/month) | 0.005<br>[-0.002, 0.01] | -0.02<br>[-0.02, -0.01] | -0.02<br>[-0.03, -0.01] |
| **Putamen** | **$CLN5^{+/-}$** | **$CLN5^{-/-}$** | **Difference** |
| Volume at 5m ($cm^3$) | 0.9<br>[0.9, 1] | 0.9<br>[0.7, 1.1] | 0.007<br>[-0.2, 0.2] |
| Volume at 18m ($cm^3$) | 0.8<br>[0.6, 0.9] | 0.7<br>[0.8, 1] | 0.1<br>[-0.01, 0.2] |
| Slope ($cm^3$/month) | -0.007<br>[-0.01, -0.0007] | -0.004<br>[-0.009, 0.002] | 0.003<br>[-0.005, 0.01] |
| **Thalamus** | **$CLN5^{+/-}$** | **$CLN5^{-/-}$** | **Difference** |
| Volume at 5m ($cm^3$) | 1.8<br>[1.6, 2.1] | 1.8<br>[1.6, 2.1] | -0.02<br>[-0.3, 0.2] |
| Volume at 18m ($cm^3$) | 1.9<br>[1.8, 2.1] | 1.8<br>[1.6, 2] | -0.2<br>[-0.4, 0.08] |
| Slope ($cm^3$/month) | 0.009<br>[0.003, 0.02] | -0.003<br>[-0.009, 0.003] | -0.01<br>[-0.02, -0.004] |

Numbers in square brackets indicate the 95% confidence intervals.
Both volume difference and slope difference (column 3) are considered significant if the 95% confidence interval does not include zero.

**Supplementary Table 2. Linear mixed effects regression model comparing rate of change in volume between control and CLN6 affected sheep.**

| **Primary motor cortex** | **$CLN6^{+/-}$** | **$CLN6^{-/-}$** | **Difference** |
|---|---|---|---|
| Volume at 5m ($cm^3$) | 0.9<br>[0.8, 1] | 0.7<br>[0.6, 0.9] | -0.1<br>[-0.2, -0.03] |
| Volume at 18m ($cm^3$) | 0.9<br>[0.8, 1] | 0.5<br>[0.3, 0.7] | -0.4<br>[-0.5, -0.3] |
| Slope ($cm^3$/month) | 0.004<br>[-0.0002, 0.008] | -0.02<br>[-0.02, -0.01] | -0.02<br>[-0.03, -0.02] |
| **Primary sensory cortex** | **$CLN6^{+/-}$** | **$CLN6^{-/-}$** | **Difference** |
| Volume at 5m ($cm^3$) | 1.1<br>[0.9, 1.3] | 0.9<br>[0.8, 0.9] | -0.2<br>[-0.4, -0.05] |
| Volume at 18m ($cm^3$) | 1.1<br>[1, 1.2] | 0.5<br>[0.3, 0.7] | -0.6<br>[-0.7, -0.5] |
| Slope ($cm^3$/month) | 0.0006<br>[-0.005, 0.006] | -0.03<br>[-0.03, -0.02] | -0.03<br>[-0.04, -0.02] |
| **Parieto-occipital cortex** | **$CLN6^{+/-}$** | **$CLN6^{-/-}$** | **Difference** |
| Volume at 5m ($cm^3$) | 1.4<br>[1.2, 1.7] | 1<br>[0.8, 1.2] | -0.4<br>[-0.7, -0.1] |
| Volume at 18m ($cm^3$) | 1.5<br>[1.2, 1.7] | 0.6<br>[0.4, 0.8] | -0.9<br>[-1.1, -0.6] |
| Slope ($cm^3$/month) | 0.001<br>[-0.008, 0.01] | -0.03<br>[-0.04, -0.02] | -0.03<br>[-0.05, -0.02] |
| **Orbital gyrus** | **$CLN6^{+/-}$** | **$CLN6^{-/-}$** | **Difference** |
| Volume at 5m ($cm^3$) | 0.7<br>[0.6, 0.8] | 0.6<br>[0.4, 0.8] | -0.04<br>[-0.2, 0.1] |
| Volume at 18m ($cm^3$) | 0.7<br>[0.7, 0.8] | 0.4<br>[0.3, 0.6] | -0.3<br>[-0.4, -0.2] |
| Slope ($cm^3$/month) | 0.002<br>[-0.003, 0.008] | -0.02<br>[-0.02, -0.01] | -0.02<br>[-0.03, -0.01] |
| **Orbitofrontal gyrus** | **$CLN6^{+/-}$** | **$CLN6^{-/-}$** | **Difference** |
| Volume at 5m ($cm^3$) | 1.5<br>[1.3, 1.6] | 1.2<br>[1.1, 1.3] | -0.2<br>[-0.4, -0.08] |
| Volume at 18m ($cm^3$) | 1.5<br>[1.4, 1.6] | 0.8<br>[0.4, 1.1] | -0.7<br>[-0.9, -0.6] |
| Slope ($cm^3$/month) | 0.002<br>[-0.004, 0.009] | -0.04<br>[-0.05, -0.03] | -0.04<br>[-0.05, -0.03] |
| **Cerebellum** | **$CLN6^{+/-}$** | **$CLN6^{-/-}$** | **Difference** |
| Volume at 5m ($cm^3$) | 4.1<br>[3.7, 4.6] | 3.8<br>[3.1, 4.5] | -0.3<br>[-0.9, 0.3] |
| Volume at 18m ($cm^3$) | 4.2<br>[3.7, 4.7] | 4.4<br>[3.6, 5.2] | 0.2<br>[-0.4, 0.9] |
| Slope ($cm^3$/month) | 0.001<br>[-0.02, 0.02] | 0.04<br>[0.02, 0.07] | 0.04<br>[0.007, 0.07] |

| **Entolateral gyrus** | **CLN6$^{+/-}$** | **CLN6$^{-/-}$** | **Difference** |
|---|---|---|---|
| Volume at 5m (cm$^3$) | 0.6<br>[0.5, 0.7] | 0.4<br>[0.2, 0.6] | -0.2<br>[-0.4, -0.07] |
| Volume at 18m (cm$^3$) | 0.6<br>[0.7, 0.5] | 0.3<br>[0.2, 0.3] | -0.4<br>[-0.5, -0.3] |
| Slope (cm$^3$/month) | -0.0005<br>[-0.005, 0.004] | -0.01<br>[-0.02, -0.005] | -0.009<br>[-0.02, -0.002] |
| **Lateral gyrus** | **CLN6$^{+/-}$** | **CLN6$^{-/-}$** | **Difference** |
| Volume at 5m (cm$^3$) | 1.3<br>[1, 1.5] | 0.8<br>[0.6, 1.1] | -0.4<br>[-0.7, -0.2] |
| Volume at 18m (cm$^3$) | 1.3<br>[1.1, 1.5] | 0.5<br>[0.3, 0.7] | -0.8<br>[-1, -0.6] |
| Slope (cm$^3$/month) | 0.002<br>[-0.008, 0.1] | -0.02<br>[-0.04, -0.01] | -0.03<br>[-0.04, -0.01] |
| **Occipital lobe** | **CLN6$^{+/-}$** | **CLN6$^{-/-}$** | **Difference** |
| Volume at 5m (cm$^3$) | 1.2<br>[1.1, 1.3] | 0.8<br>[0.8, 0.9] | -0.4<br>[-0.5, -0.3] |
| Volume at 18m (cm$^3$) | 1.3<br>[1.1, 1.4] | 0.5<br>[0.3, 0.8] | -0.7<br>[-0.9, -0.6] |
| Slope (cm$^3$/month) | 0.006<br>[-0.0004, 0.01] | -0.02<br>[-0.03, -0.02] | -0.03<br>[-0.04, -0.02] |
| **Caudate nucleus** | **CLN6$^{+/-}$** | **CLN6$^{-/-}$** | **Difference** |
| Volume at 5m (cm$^3$) | 1.3<br>[1.2, 1.4] | 1.3<br>[1.2, 1.5] | 0.04<br>[-0.08, 0.2] |
| Volume at 18m (cm$^3$) | 1.4<br>[1.3, 1.5] | 1.2<br>[0.9, 1.5] | -0.2<br>[-0.4, 0.006] |
| Slope (cm$^3$/month) | 0.007<br>[0.002, 0.01] | -0.01<br>[-0.02, -0.007] | -0.02<br>[-0.03, -0.01] |
| **Putamen** | **CLN6$^{+/-}$** | **CLN6$^{-/-}$** | **Difference** |
| Volume at 5m (cm$^3$) | 0.8<br>[0.7, 0.9] | 0.8<br>[0.6, 0.9] | 0.02<br>[-0.1, 0.1] |
| Volume at 18m (cm$^3$) | 0.7<br>[0.7, 0.8] | 0.7<br>[0.6, 0.7] | -0.08<br>[-0.2, 0.01] |
| Slope (cm$^3$/month) | -0.001<br>[-0.006, 0.04] | -0.008<br>[-0.01, -0.003] | -0.007<br>[-0.01, 0.0006] |
| **Thalamus** | **CLN6$^{+/-}$** | **CLN6$^{-/-}$** | **Difference** |
| Volume at 5m (cm$^3$) | 1.5<br>[1.4, 1.7] | 1.5<br>[1.4, 1.6] | -0.02<br>[-0.2, 0.1] |
| Volume at 18m (cm$^3$) | 1.7<br>[1.6, 1.8] | 1.5<br>[1.2, 1.8] | -0.2<br>[-0.4, 0.02] |
| Slope (cm$^3$/month) | 0.01<br>[0.007, 0.02] | -0.0001<br>[-0.006, 0.006] | -0.01<br>[-0.02, -0.004] |

Numbers in square brackets indicate the 95% confidence intervals.
Both volume difference and slope difference (column 3) are considered significant if the 95% confidence interval does not include zero.

**Supplementary Table 3. Cortical and subcortical regions analysed and their corresponding annotations on the Ella et al 2017 sheep brain MRI atlas**

| Cortical regions | Atlas label and annotation | Corresponding atlas image* |
|---|---|---|
| Primary sensory cortex | (1) Anterior sygmoideus gyrus | Figure 4 |
| Cerebellum | (2) Cerebellum | Figure 4 |
| Primary visual cortex, Entolateral gyrus | (6) Entolateral gyrus | Figure 4 |
| Primary visual cortex, lateral gyrus | (8) Lateral gyrus | Figure 4 |
| Occipital lobe | (11) Occipital lobe | Figure 5 |
| Frontal cortex, orbital gyrus | (14) Orbital gyrus | Figure 4 |
| Frontal cortex, orbitofrontal gyrus | (15) Orbitofrontal gyrus | Figure 4 |
| Primary motor cortex | (22) Precruciate gyrus | Figure 4 |
| Parieto-occipital cortex | (23) Suprasylvius gyrus | Figure 4 |
| **Subcortical regions** | **Label and annotation** | **Corresponding atlas image*** |
| Caudate nucleus | (7) Caudate nucleus | Figure 11 |
| Putamen | (26) Putamen | Figure 11 |
| Thalamus | (28) Thalamus | Figure 11 |

* See atlas figures in: Ella A, Delgadillo JA, Chemineau P, Keller M. Computation of a high-resolution MRI 3D stereotaxic atlas of the sheep brain: Stereotaxic atlas of the sheep brain. *J Comp Neurol*. 2017;525(3):676-692. doi:10.1002/cne.24079
